# Both calcium-sensing receptor intracellular C-terminal domains support homodimer signaling

**DOI:** 10.1016/j.molpha.2025.100077

**Published:** 2025-09-23

**Authors:** Lenah S. Binmahfouz, Mahvash A. Goolam, Eleanor Barker, Arthur D. Conigrave, Donald T. Ward

**Affiliations:** 1 Department of Pharmacology and Toxicology, Faculty of Pharmacy, King Abdulaziz University, Jeddah, Saudi Arabia; 2 School of Life and Environmental Sciences, Charles Perkins Centre (D17), University of Sydney, New South Wales, Australia; 3 Faculty of Biology, Medicine and Health, The University of Manchester, United Kingdom

**Keywords:** Calcium-sensing receptor, Homodimeric G protein–coupled receptor signaling, G protein–coupled receptor *trans*-activation

## Abstract

Extracellular calcium-sensing receptors (CaSRs) are required for whole-body calcium homeostasis and operate as homodimers, shown structurally to bind only one heterotrimeric G protein at a time. Whether the intracellular domain (ICD) of the other, uncoupled monomer is functionally redundant or still required for optimal CaSR signaling remains unknown. Cotransfection of human embryonic kidney 293 cells with receptors containing both extracellular (CaSR_1_^S170A^) and intracellular (CaSR_2_^F801A^) mutations, which are nonfunctional when transfected individually, partially rescues function via *trans*-activation in CaSR_1_^S170A^:CaSR_2_^F801A^ heterodimers. Further, mutation of an inhibitory, intracellular protein kinase C site T888 permits gain-of-function in CaSR. Therefore, we disabled phosphorylation of this residue (CaSR^T888A^) by mutating one, and then both monomers in the CaSR_1_^S170A^:CaSR_2_^F801A^*trans*-activation–enabled heterodimers. The extracellular Ca^2+^–induced intracellular Ca^2+^ mobilization elicited by CaSR_1_^S170A^:CaSR_2_^F801A^ was significantly enhanced by disabling one T888 inhibitory phosphorylation site in either CaSR_1_^S170A/T888A^:CaSR_2_^F801A^ or CaSR_1_^S170A^:CaSR_2_^F801A/T888A^, and further enhanced in cells in which both T888 sites had been removed in CaSR_1_^S170A/T888A^:CaSR_2_^F801A/T888A^. The results demonstrate that both ICDs of CaSR dimers contribute independently to receptor signaling. Further, in the presence of *N*-(3-[2-chlorophenyl]propyl)-(*R*)-alpha-methyl-3-methoxybenzylamine (NPS R-568; CaSR positive allosteric modulator), extracellular Ca^2+^–stimulated signaling from the nonfunctional CaSR^S170A^ mutant was rescued to wild-type levels by removal of the inhibitory T888 phosphorylation site in CaSR^S170A/T888A^ homodimers. Therefore, although CaSR^S170A^ destabilizes the closed (active) form of the Venus fly trap in wild-type homodimers, receptor function can be rescued by relieving intracellular C-terminal domain–dependent inhibition of signaling.

**Significance Statement:**

Despite the homodimeric calcium-sensing receptor coupling to only one G protein at a time, disinhibiting both intracellular domains elicits a greater increase in intracellular Ca^2+^ mobilization than disinhibiting only one, suggesting a positive functional contribution from both subunits.

## 1 Introduction

The calcium-sensing receptor (CaSR) is a key controller of mammalian extracellular Ca^2+^ (Ca^2+^_o_) homeostasis and acts by suppressing parathyroid hormone secretion and renal Ca^2+^ reabsorption. CaSRs form stable disulfide-linked homodimers in the endoplasmic reticulum early in their protein maturation and signal as constitutive dimers.^1^, ^2^, ^3^, ^4^, ^5^, ^6^ CaSR signaling is highly influenced by protein kinase C (PKC)–mediated phosphorylation of T888 in the receptor’s intracellular C-terminal domain (ICD),^7^^,^^8^ and the mutations T888A and T888M, which disable phosphorylation, are activating in vitro. In support of this, T888M has been linked to hypocalcemia in a kindred with autosomal dominant hypocalcemia.^9^ Upon receptor activation, it is not known whether T888 phosphorylation is restricted to one subunit at a time, eg, dependent upon the subunit which is engaged in G protein binding. In addition, it is not known whether the receptor-activating effect of T888 dephosphorylation is greater when only one or both subunits are dephosphorylated.

Bai et al^10^ reported that coexpression of 2 nonfunctioning CaSR mutants could complement one another to partially rescue receptor function, leading them to conclude that the receptors normally operate as functional dimers. In addition, they proposed a so-called *trans-*activation mechanism, by which the receptor appeared to transmit the signal from the wild-type (WT) extracellular domain (ECD) of one subunit to the WT 7-TMD (7-transmembrane domain) and ICD of the neighboring subunit.^10^ The phenomenon of subunit complementation in cells cotransfected with selected mutant receptor subunits has been confirmed for the human CaSR^11^^,^^12^ and also for the rat CaSR.^13^ Experimentally, this is useful because when 2 such nonfunctional mutants are cotransfected, rescued signaling can only come from heterodimers and not from the nonfunctional homodimers.

X-ray crystal structures of the CaSR have confirmed that isolated ECDs are homodimers,^3^^,^^14^ whereas recently solved cryo-EM structures of the full-length CaSR in complex with several G proteins, including G_q_, G_i1_, G_i3_, and G_s_, indicate that upon activation, only one heterotrimeric G protein binding site forms in one subunit of receptor dimers.^15^^,^^16^ These observations raise the possibility that the conformation of the active receptor complex in WT homodimers returns to a neutral inactive symmetric dimer configuration^4^ before adopting the next active conformation. Whether receptor phosphorylation/dephosphorylation contributes to the activation sequence, and if so, how, is unknown.

Thus, in seeking to determine the role of T888 phosphorylation/dephosphorylation in CaSR function, we decided to focus on mutant heterodimers that exhibit subunit complementation using one subunit that bears a nonfunctioning mutation in its ECD ligand-sensing region and a second subunit that bears a nonfunctioning mutation in its 7-TMD G protein–coupling region. In particular, we asked the following questions: (1) Does the nonphosphorylatable mutant T888A selectively promote receptor function when introduced into one but not the other subunit? And (2) does the introduction of T888A into both subunits more powerfully promote receptor function?

Therefore, the aims of this study were to explore the impacts of the nonphosphorylatable CaSR mutant T888A in the receptor’s ICDs either singly or together on the function of specifically designed mutant receptor dimers as reported by Ca^2+^_o_-induced G_q/11_-dependent intracellular Ca^2+^ (Ca^2+^_i_) mobilization. The receptor dimers were designed such that one subunit bearing S170A was defective in its ability to support Venus fly trap (VFT) closure, and the other subunit bearing F801A was defective in its ability to support G protein binding.^13^^,^^15^^,^^16^

## 2 Materials and methods

### 2.1 Cell culture

Human embryonic kidney (HEK)–293 cells (CRL-1573; ATCC) were cultured in Dulbecco’s modified Eagle’s medium (DMEM) supplemented with 10% (v/v) heat-inactivated FBS (Sigma-Aldrich) in a 5% CO_2_ incubator at 37 °C. The HEK-293 cells were shown to be free from mycoplasma by in-house PCR testing.

### 2.2 Generation of CaSR mutants, DNA synthesis, and transfections

Site-directed mutagenesis was employed using the WT human CaSR to generate CaSR_1_^S170A^, CaSR_2_^F801A^, CaSR^T888A^, CaSR_1_^S170A/T888A^, and CaSR_2_^F801A/T888A^ mutants using the Quik-Change lightning site-directed mutagenesis kit (Agilent Technologies Ltd) with the human pcDNA3.1/Hygromycin CaSR vector used as the template. All mutations were verified by DNA sequencing (The University of Manchester in-house service), aligned to the hCaSR sequence (GenBank accession no U20759) using MEGA-X software. For the preparation of double mutants, the DNA containing one of the single point mutations was used as a template. The CaSR-GABA_B2_ plasmid was generated by appending residues 760 to 941 of the human GABA_B2_ C-tail onto CaSR truncation mutant 909X. A variant of this construct, CaSR-GABA_B2_(_KKTNX_), was also generated in which the GABA_B2_ C-tail was truncated after residue 820, and the intracellular retention motif, ”KKTN” was inserted immediately prior to the stop codon. After digestion of template DNAs with DpnI, WT and mutant plasmids were transformed into *Escherichia coli*, and DNA synthesis and purification were undertaken using midi-preps as described previously.^17^

HEK-293 cells were transfected at 75% to 95% confluency with 1 *μ*g lots of WT or mutant CaSRs per well in 6-well plates using FuGENE 6 according to the manufacturer’s instructions. For cotransfections, we used 1 *μ*g lots of both required plasmid DNAs (total DNA amount 2 *μ*g). Cells were then cultured for a further 48 hours at 37 °C in DMEM supplemented with 10% (v/v) heat-inactivated FBS (Sigma-Aldrich) prior to signaling analyses.

#### 2.2.1 Assays of total and surface expression

Assays of total and surface expression were based on the method of Stepanchick et al.^18^ Briefly, HEK-293 cells were transiently transfected with pcDNA3.1 empty vector controls or N-terminal FLAG-tagged WT or mutant CaSRs using XtremeGENE HP transfection reagent according to the manufacturer’s instructions for 18 to 24 hours. The N-terminal FLAG tag was located between CaSR residues 371 and 372 in a functionally silent loop of the ECD as described previously.^17^ Cells were then detached with Trypsin-EDTA and pelleted by centrifugation for 2 minutes at 360*g* (Eppendorf S-4-104 rotor; radius 19 cm; 1300 rpm). The supernatants were discarded, and the pellets were resuspended in 2 mL fresh DMEM (10% FBS). Suspensions were then plated in 0.1 mL aliquots in poly-D-lysine–coated 96-well plates for 24 hours. The cells in individual wells were then washed once with ice-cold PBS and fixed by incubation with 0.1 mL 4% paraformaldehyde on ice for 5 minutes. Samples to be assayed for surface expression remained in 4% paraformaldehyde, whereas samples to be assayed for total expression were permeabilized by the addition of 0.1 mL methanol on ice for 15 minutes. Subsequent steps were all performed at room temperature. All samples were washed once with Tris-buffered saline/Tween 20 (TBST; 15 mM Tris [pH 8], 150 mM NaCl, 0.1% (v/v) Tween 20) and blocked with 1% milk in TBST blocking solution for 1 hour. Following blocking, the cells were washed once with TBST, then incubated for 1 hour in either 0.1 mL anti-FLAG horseradish peroxidase–conjugated antibody (Sigma-Aldrich; cat #A8592), diluted 1:5000 in TBST, or 0.1 mL TBST for no-antibody controls. Cell samples were washed 3 times with TBST and the enzyme substrate 3,3',5,5'-tetramethybenzidine (Sigma-Aldrich; cat #T0440) was then added, and the incubations were continued for 8 minutes in the dark. Reactions were stopped by the addition of 80 *μ*L 1 M HCl. For all samples, 140 *μ*L lots were transferred to the wells of a fresh 96-well plate, and the absorbances were measured at 450 nm in a Tecan Infinite M1000 Pro microplate reader.

### 2.3 Intracellular calcium imaging

Cells were cultured on 16 mm glass coverslips for 24 to 48 hours in growth media. Fura-2-acetoxymethyl ester (Fura-2AM, Life Technologies), a fluorescent Ca^2+^_i_ indicator, was dissolved at 1 *μ*M in 0.1% (v/v) DMSO. Coverslips were washed with experimental buffer containing 125 mM NaCl, 1.2 mM CaCl_2_, 0.5 mM MgCl_2_, 4 mM KCl, 5.5 mM glucose, 0.1% (w/v) bovine serum albumin, and 20 mM HEPES, pH 7.4. Cells on coverslips were then loaded with Fura-2AM in the same experimental buffer for 1 hour in the dark at room temperature. When increasing the CaCl_2_ concentration in the buffer, the NaCl concentration was reduced to maintain isotonicity. The coverslips were then mounted in a perfusion chamber (Warner Instruments) and continuously perfused at room temperature.

The Fura-2AM–loaded cells were visualized using a Nikon Diaphot inverted microscope through a 40× oil-immersion objective lens equipped with a digital camera charge-coupled device. Briefly, the computer-controlled filter wheel allowed alternation between the 350 and 380 nm excitation wavelengths, and ratios of the 2 fluorescent signals were acquired every 2 seconds. The fluorescence images were captured with a coupled device camera connected to the computer. Experiments included measuring fluorescence intensities with experimental buffers containing different concentrations of Ca^2+^ alone or Ca^2+^ supplemented with a CaSR agonist, *N*-(3-[2-chlorophenyl]propyl)-(*R*)-alpha-methyl-3-methoxybenzylamine (NPS R-568) to measure the relative Ca^2+^_o_ sensitivities of the receptors.

### 2.4 Data and statistical analysis

The software MetaFluor was used for recording and analyzing the changes in Ca^2+^_i_ during the imaging experiments. In order to determine the relative changes in Fura-2AM ratios, the area under the curve for all the cells in the field of view “global” was calculated for each concentration using GraphPad Prism (version 8) with the baseline ratio (0.5 mM Ca^2+^) subtracted from this. The area under the curve was presented graphically as mean ± SD percent of the maximum response. Sigmoidal concentration-effect curves with variable slope were generated with GraphPad Prism using nonlinear regression analysis. All data were screened for statistical distribution using the Shapiro-Wilk normality test, and statistical significance was determined by performing either Student’s paired/unpaired *t* test, one-way ANOVA for multiple comparisons, followed by Tukey**/**Dunnett’s post hoc test, or by *F* test. *P* values of <.05 were considered significantly different. Due to the exploratory nature of the study, *P* values cannot be interpreted as hypothesis-testing, but only as descriptive.

## 3 Results

Unmodified, the complementary mutant heterodimer CaSR^S170A^:CaSR^F801A^ cannot be used to investigate the dimeric receptor’s requirements for intracellular C-terminal domains. However, there is a well defined phospho-regulatory site at T888 in each of the 2 proximal C termini. These sites mediate tonic inhibition of CaSR-mediated signaling and can be selectively disabled by the introduction of the mutant T888A into one or both subunits. We therefore introduced T888A into CaSR_1_^S170A^ (to generate CaSR_1_^S170A/T888A^) and CaSR_2_^F801A^ (to generate CaSR_2_^F801A/T888A^) and investigated their impacts in the following mutant heterodimers: (1) CaSR_1_^S170A/T888A^:CaSR_2_^F801A^; (2) CaSR_1_^S170A^:CaSR_2_^F801A/T888A^; and (3) CaSR_1_^S170A/T888A^:CaSR_2_^F801A/T888A^. Thus, we were able to test the effects of T888A alone, on a background of either CaSR_1_^S170A^ or CaSR_2_^F801A^, or in combination.

**Fig. 1:**
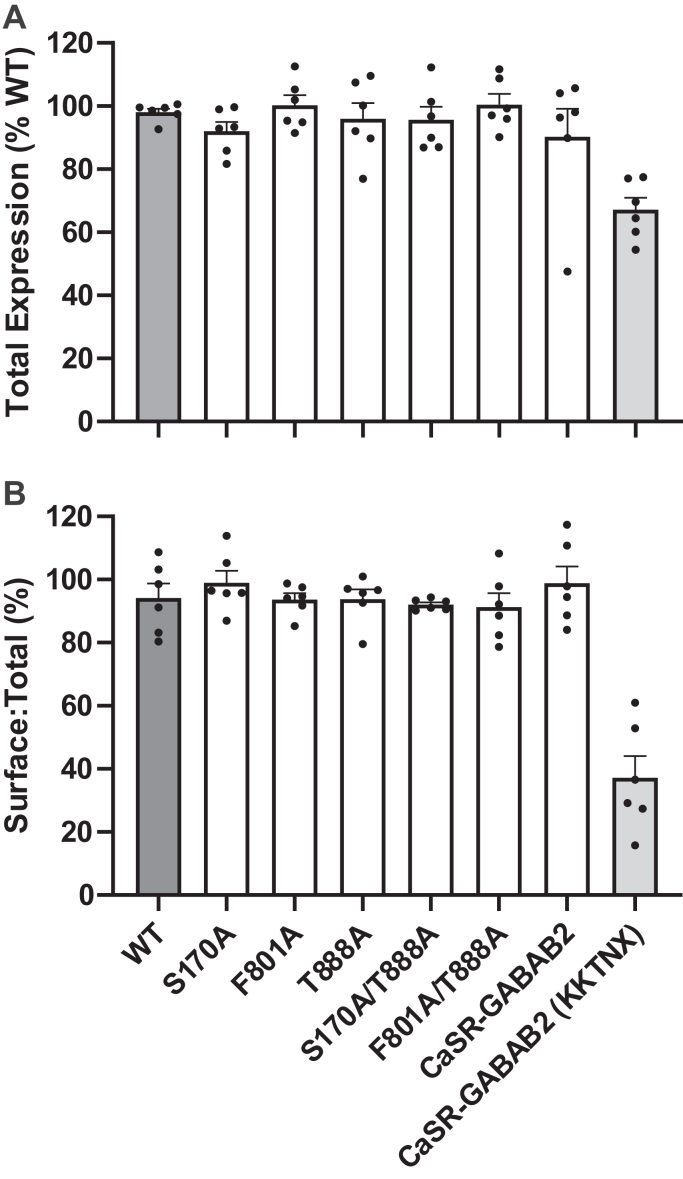
CaSR mutants exhibit equivalent (A) total levels of expression and (B) cell surface expression. HEK-293 cells were transiently transfected with pcDNA3.1, CaSR^WT^, CaSR_1_^S170A^, CaSR_2_^F801A^, CaSR^T888A^, CaSR_1_^S170A/T888A^, CaSR_2_^F801A/T888A^, CaSR-GABA_B2_, or CaSR-GABAB2(_KKTN_X). Transfected cells were fixed, and the samples analyzed for total expression were then permeabilized. Cell surface expression and total expression were determined through an ELISA. Data are represented as mean ± SEM, calculated from 18 readings per condition, from 6 independent biological repeats. (B) Cell surface expression was significantly reduced in the CaSR-GABA_B2_(_KKTN_X) positive control (two-way ANOVA; ∗∗∗*P* < .001). (A) Total protein was significantly reduced in the pcDNA3.1 template control and CaSR-GABA_B2_(_KKTN_X) positive control, relative to CaSR^WT^ (one-way ANOVA; ∗∗∗ *P* < .001 and ∗∗*P* < .01, respectively).

We first confirmed equivalent surface expression of the various mutants. HEK-293 cells were transfected with cDNAs for the vector-only pcDNA3.1 control, the CaSR^WT^, or one or more CaSR mutants as required. For the determination of total and surface expression by ELISAs, each CaSR cDNA construct was labeled with an N-terminal FLAG peptide between residues 371 and 372 in a functionally silent loop in the ECD. We found no differences between the total expressions of any of the mutant subunits tested including S170A, F801A, T888A, S170A/T888A, F801A/T888, and the control construct CaSR-GABA_B2_, when compared with WT (one-way ANOVA; *P* = .60; n = 6 per group; Fig. 1A). After correction for the background levels measured in cells transfected with pcDNA3.1, the WR CaSR exhibited a surface: total expression ratio of 94.2 ± 5.0 (n = 6) and comparable results were obtained for all other CaSR constructs tested, with the exception of CaSR-GABA_B2_ (KKTNX), which contained an endoplasmic reticulum retention motif KKTN at its C terminus prior to the stop codon (Fig. 1B). Thus, CaSR-GABA_B2_ (KKTNX) exhibited a markedly reduced surface: total expression value of 37.1% ± 7.6% (n = 6). With respect to the surface: total expression ratio, one-way ANOVA demonstrated no differences between the WT CaSR and any of the CaSR mutants/double mutants tested in the study, with a range of 91.3% to 98.9% (*P* = .66). Thus, any differences in signaling responses between cells transfected with the mutant constructs of primary interest to the study could be assigned to differences in function and not to differences in either total or surface expression.

### 3.1 Functional complementation in mutant heterodimers

**Fig. 2:**
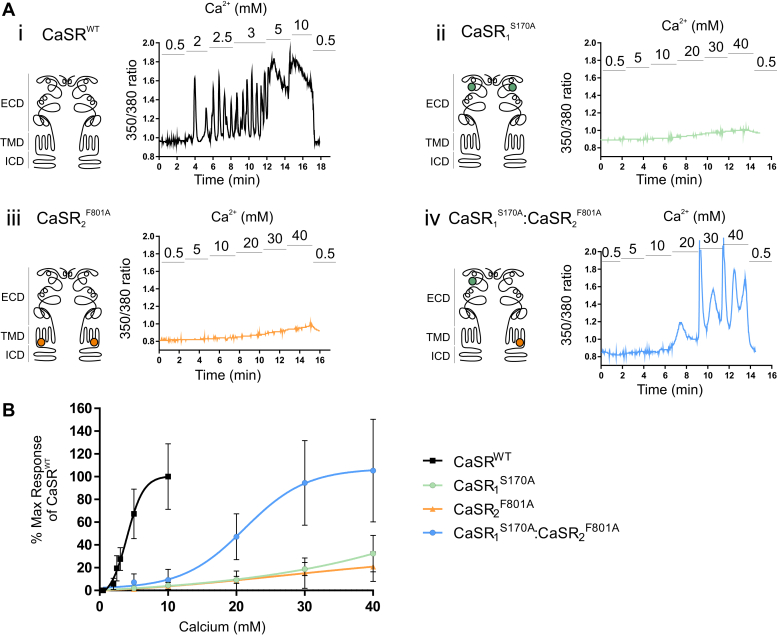
Cotransfection of inactive CaSR receptor mutants partially rescues Ca^2+^_i_ mobilization in HEK-293 cells. HEK-293 cells were transiently transfected with (i) CaSR^WT^, (ii) CaSR_1_^S170A^, (iii) CaSR_2_^F801A^, and (iv) CaSR_1_^S170A:F801A^. After 48 hours, cells were loaded with Fura-2AM, then exposed to 0.5 to 40 mM Ca^2+^_o_, and the resulting changes in Ca^2+^_i_ mobilization were measured. (A) Representative Ca^2+^_i_ mobilization traces (Fura-2AM ratio) from a single cell in response to calcium. The schematic diagrams of the CaSR shown on the left of each trace demonstrate the location of each mutant in either the ECD, transmembrane domain (TMD), or the intracellular domain (ICD). (B) The concentration-effect curves showing a significant difference between log EC_50_ values for Ca^2+^_o_ in the CaSR^WT^ vs CaSR_1_^S170A:F801A^. ∗∗∗*P* < .001 by unpaired *t* test. Data are collected from 3 to 6 coverslips and are represented as mean ± SD, as a percentage of the maximum responses of the CaSR^WT^ at 10 mM Ca^2+^_o_.

We first sought to confirm the lack of function of homodimers formed separately from CaSR_1_^S170A^ and CaSR_2_^F801A^ and to demonstrate the functional competence of CaSR_1_^S170A^:CaSR_2_^F801A^ heterodimers. HEK-293 cells were transfected with CaSR_1_^S170A^ and CaSR_2_^F801A^ either singly or together as described in Materials and Methods. As previously described,^19^ Fura-2AM–loaded HEK-293 cells that were transfected with the WT CaSR were quiescent at Ca^2+^_o_ levels at or below 0.5 mM but exhibited repetitive Ca^2+^_i_ oscillations at Ca^2+^_o_ levels between 2 and 3 mM; at higher Ca^2+^_o_ concentrations (5 or 10 mM), they exhibited sustained elevations in Ca^2+^_i_ (Fig. 2Ai). Fura-2AM–loaded HEK-293 cells that were transfected with either CaSR_1_^S170A^ or CaSR_2_^F801A^ alone, however, failed to exhibit Ca^2+^_o_-stimulated Ca^2+^_i_ responses above slow, low-amplitude shifts in baseline Ca^2+^_i_ (Fig. 2, Aii and Aiii) that were also observed in untransfected HEK-293 cells, which do not express the CaSR (data not shown). This modest, slow increase is likely to result from nonreceptor mediated Ca^2+^ influx.^20^

HEK-293 cells that were cotransfected with CaSR_1_^S170A^ and CaSR_2_^F801A^, however, exhibited a partial rescue of receptor function arising presumably from CaSR_1_^S170A^:CaSR_2_^F801A^ heterodimers. Compared to cells transfected with CaSR^WT^, we observed a rightward shift in the Ca^2+^_o_ concentration-response curve (Fig. 2B) and an associated increase in EC_50_ for Ca^2+^_o_ (21.4 mM vs 4.5 mM, respectively; *P* < .001 for comparison between pEC_50_ values). Although this behavior has been previously identified as *trans-*activation signaling based on the apparent pathway of receptor activation (from the WT ECD of one subunit to the WT 7-TMD unit of the other), it seems feasible that the functional complementation occurs at 2 interacting levels in these mutant receptor dimers: (1) in the dimeric VFT domains; and (2) in the dimeric 7-TMDs.

### 3.2 Functional impact of an ICD mutant T888A that disables PKC-dependent phosphorylation

**Fig. 3:**
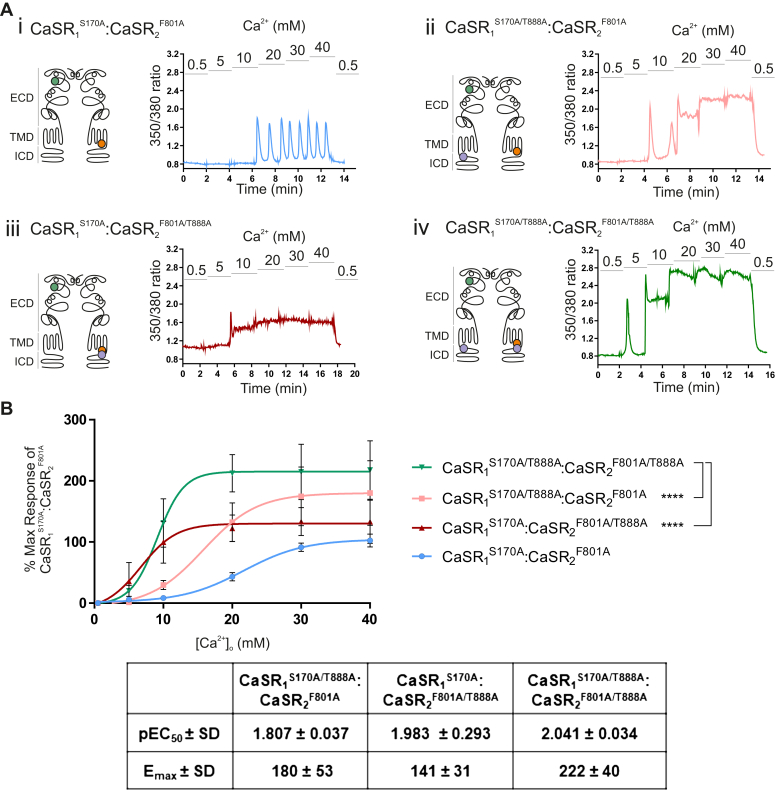
Enhancement of CaSR-induced Ca^2+^_i_ mobilization of heterodimers containing the T888A mutation in one or both subunits. HEK-293 cells were transiently cotransfected with (i) CaSR_1_^S170A^ and CaSR_2_^F801A^, (ii) CaSR_1_^S170A/T888A^ and CaSR_2_^F801A^, (iii) CaSR_1_^S170A^ and CaSR_2_^F801A/T888A^, and (iv) CaSR_1_^S170A/T888A^ and CaSR_2_^F801A/T888A^ in a 1:1 ratio for 48 hours. After 48 hours, cells were loaded with Fura-2AM, then exposed to 0.5 to 40 mM Ca^2+^_o_, and the resulting changes in Ca^2+^_i_ mobilization were measured. (A) Representative Ca^2+^_i_ mobilization traces (Fura-2AM ratio) from a single cell in response to calcium. The schematic diagrams of the CaSR shown on the left of each trace demonstrate the location of each mutant in either the ECD, transmembrane domain (TMD), or the intracellular domain (ICD) in each monomer of the functional heterodimer. (B) Data are collected from 7 to 12 coverslips from 3 independent transfections. The CaSR_1_^S170A^:CaSR_2_^F801A^ (n = 3, from 2 transfections) is included for visual comparison. Data are represented as mean ± SD, as a percentage of the maximum responses of the CaSR_1_^S170A^:CaSR_2_^F801A^ heterodimer at 40 mM Ca^2+^_o_. ∗∗∗∗*P* < .0001 vs CaSR_1_^S170A/T888A^:CaSR_2_^F801A/T888A^ curve by *F* test.

The functional interaction between the ICDs of the CaSR dimer was next studied using the *trans*-activation mechanism demonstrated in Fig. 3. This study had 2 specific questions: first, whether the introduction of T888A into one or both subunits of functional heterodimers (ie, into CaSR_1_^S170A^:CaSR_2_^F801A^) would further enhance function, thereby providing evidence that both ICDs contribute independently to signaling; and second, whether the introduction of the gain-of-function mutant T888A into subunits with nonfunctional intracellular loops (ie, CaSR_2_^F801A^) might rescue *trans*-activation signaling.

Therefore, site-directed mutagenesis was employed to introduce T888A into the existing loss-of-function mutants. Depending on the choice of vectors for cotransfection, it was possible to produce 4 different CaSR heterodimers: (1) a fully PKC-inhibitable heterodimer with unaltered T888 present on both monomers (ie, CaSR_1_^S170A^:CaSR_2_^F801A^); (2) one with a single T888A mutation in the functional ICD subunit (ie, CaSR_1_^S170A/T888A^); (3) one with a single T888A mutation in the nonfunctional ICD subunit (ie, CaSR_2_^F801A/T888A^); and (4) a heterodimer with T888A mutations on the ICDs of both mutant subunits (ie, CaSR_1_^S170A/T888A^:CaSR_2_^F801A/T888A^).

In order to characterize the functional interaction between the ICDs, Ca^2+^_i_ mobilization was quantified in these 4 resulting CaSR heterodimers of interest after stimulation with increasing Ca^2+^_o_ concentrations (0.5 mM to 40 mM, Fig. 3). As expected, the introduction of T888A into the subunit with a functional ICD to form CaSR_1_^S170A/T888A^:CaSR_2_^F801A^ heterodimers significantly enhanced Ca^2+^_o_-stimulated Ca^2+^_i_ mobilization (*P* < .001 by *F* test; Fig. 3, Aii and B) with the EC_50_ for Ca^2+^_o_ reduced from 22.0 to 15.7 mM relative to CaSR_1_^S170A^:CaSR_2_^F801A^ heterodimers. Similarly, CaSR_1_^S170A^:CaSR_2_^F801A/T888A^ heterodimers (containing T888A in the subunit with nonfunctional ICDs) also exhibited significantly enhanced Ca^2+^_o_-induced Ca^2+^_i_ mobilization (*P* < .001 by *F* test; Fig. 3, Aiii and B) with the EC_50_ for Ca^2+^_o_ decreased from 22.0 mM for CaSR_1_^S170A^:CaSR_2_^F801A^ to 7 mM for CaSR_1_^S170A^:CaSR_2_^F801A/T888A^. Crucially, when T888A was present on the intracellular C termini of both heterodimers (CaSR_1_^S170A/T888A^:CaSR_2_^F801A/T888A^), the gain-of-function was additive as revealed by a further enhancement of Ca^2+^_o_ potency (*P* < .001 by *F* test vs the mutant heterodimers containing only one T888A). The increased Ca^2+^_i_ mobilization seen with the CaSR_1_^S170A/T888A^:CaSR_2_^F801A/T888A^ heterodimer resulted mostly from an apparent decrease in EC_50_ for Ca^2+^_o_ versus CaSR_1_^S170A/T888A^:CaSR_2_^F801A^ (9 mM vs 15.7 mM, respectively) and by an apparent increase in maximum effect (E_max_) for Ca^2+^_o_ versus CaSR_1_^S170A/T888A^:CaSR_2_^F801A/T888A^ (222% vs 141%, respectively; Fig. 3B).

Therefore, this indicates that the signal cannot originate entirely from a single monomer (despite the receptor coupling to just one G protein at a time), as there should not be a further enhancement of signal when the T888A mutation is present on both monomers as opposed to just one. Together, these findings indicate that both ICDs contribute to dimeric CaSR signaling and Ca^2+^_o_ sensitivity, despite the coupling of only a single G protein.

### 3.3 The effect of T888A on mutant CaSR homodimers

**Fig. 4:**
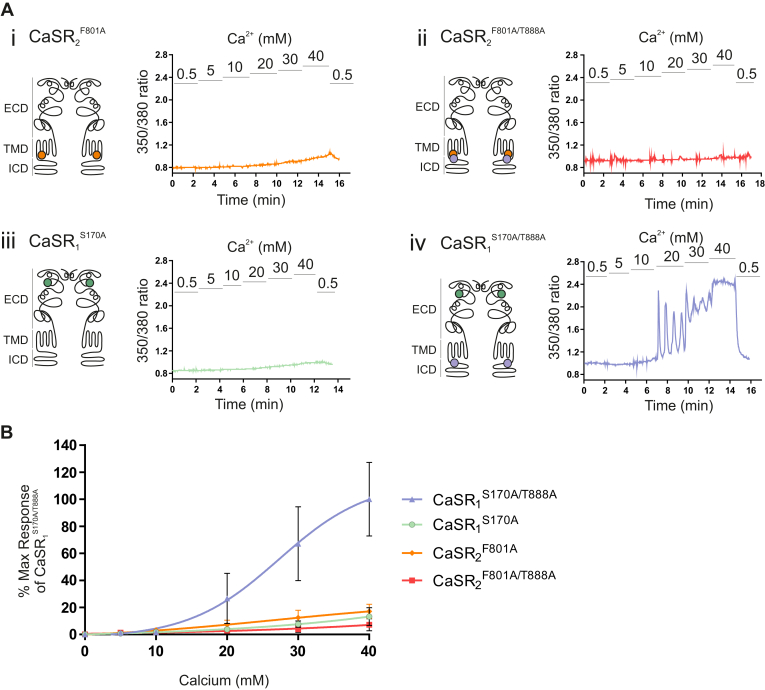
The CaSR_1_^S170A/T888A^ homodimer partially rescues Ca^2+^_i_ mobilization. HEK-293 cells were transiently transfected with the following (homodimer) mutants: (i) CaSR_2_^F801A^, (ii) CaSR_2_^F801A/T888A^, (iii) CaSR_1_^S170A^, and (iv) CaSR_1_^S170A/T888A^. After 48 hours, cells were loaded with Fura-2AM, then exposed to 0.5 to 40 mM Ca^2+^_o_, and the resulting changes in Ca^2+^_i_ mobilization were measured. (A) Representative Ca^2+^_i_ mobilization traces (Fura-2AM ratio) from a single cell in response to calcium. The schematic diagrams of the CaSR shown on the left of each trace demonstrate the location of each mutant in either the ECD, transmembrane domain (TMD), or the intracellular domain (ICD). (B) The concentration-effect curves show Ca^2+^_i_ mobilization responses only with CaSR_1_^S170A/T888A^. Data were collected from 4 to 7 coverslips. Data are represented as mean ± SD, as a percentage of the maximum responses of the CaSR_1_^S170A/T888A^ at 40 mM Ca^2+^_o_.

We next investigated the effect of singly transfecting HEK-293 cells with CaSR_1_^S170A^ or CaSR_2_^F801A^ mutant constructs modified by the introduction of T888A. In this way, we investigated the functions of mutant homodimers formed from either CaSR_1_^S170A/T888A^ or CaSR_2_^F801A/T888A^. As observed for the experiments shown in Fig. 2, HEK-293 cells that expressed either CaSR_2_^F801A^ or CaSR_1_^S170A^ mutant homodimers were nonfunctional (Fig. 2, Ai and Aiii, respectively). Interestingly, however, HEK-293 cells that expressed CaSR_1_^S170A/T888A^ homodimers (Fig. 4Aiv) but not CaSR_2_^F801A/T888A^ homodimers (Fig. 4Aii) exhibited partial rescue of Ca^2+^_o_-induced Ca^2+^_i_ mobilization, albeit at high Ca^2+^_o_ concentrations (EC_50_ for Ca^2+^_o_ 27.2 mM, Fig. 4, Aiv and B). It should be noted that there was no response of these receptors to 10 mM Ca^2+^_o_, unlike for CaSR_1_^S170A/T888A^:CaSR_2_^F801A/T888A^, which exhibited an EC_50_ <10 mM (Fig. 3), suggesting that the finding reported in Fig. 2 cannot be explained by the rescued signal in CaSR_1_^S170A/T888A^ homodimers alone. Nevertheless, the introduction of T888A into CaSR_1_^S170A^ did partially rescue the function of CaSR_1_^S170A^ homodimers (at higher concentrations), a loss-of-function mutant that exhibits impaired closure of the dimeric VFT unit. In contrast, introduction of T888A into CaSR_2_^F801A^ failed to rescue the function of CaSR_2_^F801A^ homodimers, which were unable to mobilize Ca^2+^_i_, at any of the Ca^2+^_o_ concentrations tested up to 40 mM (Fig. 4Aii).

**Fig. 5:**
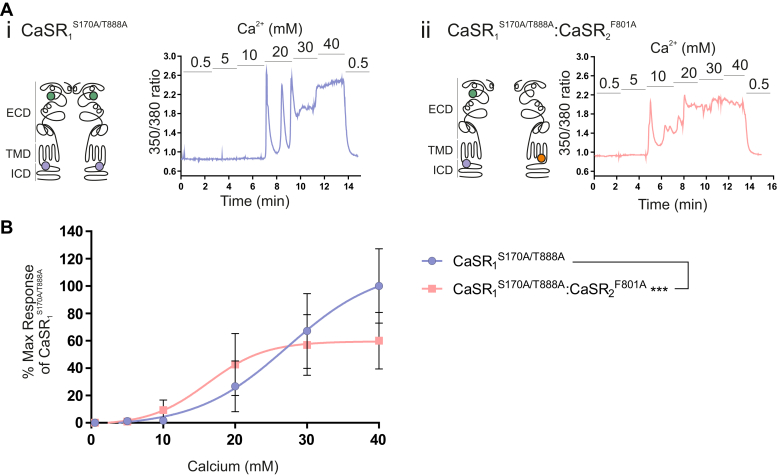
The CaSR_1_^S170A/T888A:F801A^ heterodimer is more sensitive to Ca^2+^_o_ compared to the CaSR_1_^S170A/T888A^ homodimer. HEK-293 cells were transiently transfected with either (i) CaSR_1_^S170A/T888A^ alone, or (ii) cotransfected with CaSR_1_^S170A/T888A^ and CaSR_2_^F801A^. After 48 hours, cells were loaded with Fura-2AM, then exposed to 0.5 to 40 mM Ca^2+^_o_, and the resulting changes in Ca^2+^_i_ mobilization were measured. (A) Representative Ca^2+^_i_ mobilization traces (Fura-2AM ratio) from a single cell in response to calcium. The schematic diagrams of the CaSR shown on the left of each trace demonstrate the location of each mutant in either the ECD, transmembrane domain (TMD), or the intracellular domain (ICD). (B) CaSR_1_^S170A/T888A:F801A^ heterodimer exhibited a leftward-shifted concentration-effect response relative to CaSR_1_^S170A/T888A^ homodimer with a significantly reduced EC_50_ value (unpaired *t* test; ∗∗∗*P* < .001). Data are collected from 7 to 8 coverslips. Data are represented as mean ± SD, as a percentage of the maximum responses of the CaSR_1_^S170A/T888A^ at 40 mM Ca^2+^_o_.

Finally, we determined the impact of transfecting HEK-293 cells with CaSR_2_^F801A^ as well as CaSR_1_^S170A/T888A^. We undertook these experiments to determine whether the formation of CaSR_1_^S170A/T888A^:CaSR_2_^F801A^ heterodimers, as well as CaSR_1_^S170A/T888A^ homodimers in the same cells, would impact the functional response. Interestingly, we observed enhanced Ca^2+^_o_ potency together with reduced efficacy. Thus, the E_max_ value for CaSR_1_^S170A/T888A^:CaSR_2_^F801A^ heterodimers was reduced by around 50% compared to CaSR_1_^S170A/T888A^ homodimers (*P* < .01), consistent with the loss of one G protein binding site in mutant heterodimers (Fig. 5). Interestingly, however, cells that expressed CaSR_1_^S170A/T888A^:CaSR_2_^F801A^ heterodimers exhibited enhanced Ca^2+^_o_ potency with respect to CaSR_1_^S170A/T888A^ homodimers (EC_50_ for Ca^2+^_o_ 17.5 mM vs 30.6 mM; *P* < .001, Fig. 5B) pointing to reduced resistance to the adoption of the active receptor conformation in response to Ca^2+^_o_ in the presence of T888A.

### 3.4 The effect of the positive allosteric modulator NPS R-568 on the function of mutant CaSR homodimers

**Fig. 6:**
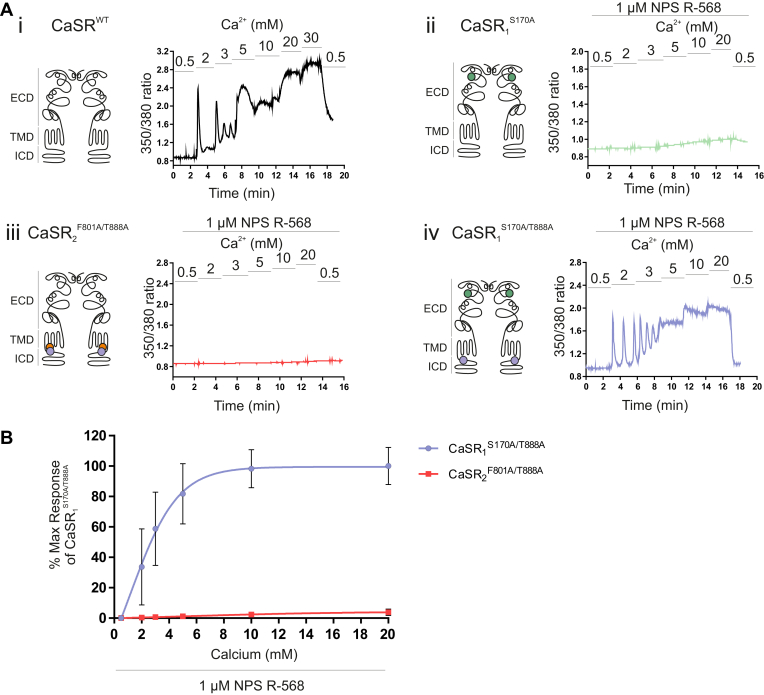
Positive allosteric modulation of the CaSR_1_^S170A/T888A^ homodimer mutant. HEK-293 cells were transiently transfected with the following: (i) CaSR^WT^, (ii) CaSR_1_^S170A^, (iii) CaSR_2_^F801A/T888A^, and (iv) CaSR_1_^S170A/T888A^. After 48 hours, cells were loaded with Fura-2AM, then exposed to 0.5 to 30 mM Ca^2+^_o_, in the presence of 1 *μ*M NPS R-568 (positive allosteric modulator), and the resulting changes in Ca^2+^_i_ mobilization were measured. (A) Representative Ca^2+^_i_ mobilization traces (Fura-2AM ratio) from a single cell in response to calcium (i) or calcium supplemented with 1 *μ*M NPS R-568 (ii–iv). The schematic diagrams of the CaSR shown on the left of each trace demonstrate the location of each mutant in either the ECD, transmembrane domain (TMD), or the intracellular domain (ICD). (B) The generated Ca^2+^_o_ concentration-effect curves for (iii) and (iv) show enhanced Ca^2+^_o_ responses only for the CaSR_1_^S170A/T888A^ mutant (pEC_50_; 2.47 ± 1.40), which are comparable to the CaSR^WT^ response (i). NPS R-568 had no effect on the CaSR_1_^S170A^ homodimer (ii). Data are collected from 4 to 5 coverslips. Data are represented as mean ± SD, as a percentage of the maximum responses of the CaSR_1_^S170A/T888A^ at 10 mM Ca^2+^_o_ supplemented with 1 *μ*M R-568.

We next investigated the effect of the positive allosteric modulator (PAM) NPS R-568 on calcimimetic enhancement of Ca^2+^_o_-stimulated Ca^2+^_i_ mobilization from the mutant CaSR homodimers CaSR_1_^S170A/T888A^ and CaSR_2_^F801A/T888A^. CaSR_2_^F801A/T888A^ exhibited no response in the absence or presence of 1 *μ*M NPS R-568 (Fig. 6Aiii). In contrast, the response of HEK-293 cells transfected with CaSR_1_^S170A/T888A^ was markedly enhanced by 1 *μ*M NPS R-568 (EC_50_ for Ca^2+^_o_ = 3.1 mM; Fig. 6, Aiv and B). Indeed, in the presence of NPS R-568, the Ca^2+^_i_ response by CaSR_1_^S170A/T888A^ was similar to that observed for the CaSR^WT^, with Ca^2+^_i_ oscillations in 2 to 3 mM Ca^2+^_o_ and sustained elevations in Ca^2+^_i_ at Ca^2+^_o_ ≥5 mM (Fig. 6Ai). Strikingly, however, NPS R-568 had no effect on the function of CaSR_1_^S170A^ homodimers in which T888 was still present (Fig. 6Aii). Taken together, the results indicate that the loss of receptor function arising downstream of the loss of stable VFT closure in CaSR_1_^S170A^ homodimers is dependent on persistent and perhaps irreversible T888 phosphorylation and can be partially restored in the presence of T888A in both subunits and fully restored in the additional presence of the PAM NPS R-568.

## 4 Discussion

Previously, Jacobsen et al^13^ reported that coexpression of 2 loss-of-function mutant subunits, CaSR_1_^S170A^ and CaSR_2_^F801A^, affecting the ECD and 7-TMD, respectively, could partially rescue CaSR function as measured by Ca^2+^_o_-induced IP_1_ accumulation. We have confirmed and extended these observations for Ca^2+^_o_-induced Ca^2+^_i_ mobilization. S170 is required for ligand-dependent VFT domain closure^3^^,^^14^ and is tightly conserved across species and in other family C of G protein–coupled receptors (GPCRs).^21^, ^22^, ^23^, ^24^ The homologous residue T188 in metabotropic glutamate receptor (mGluR)-1 supports glutamate binding in both inactive and active forms, and also supports VFT closure even if glutamate is not bound.^25^ F801, located in ICL3 of the 7-TMD unit, is also tightly conserved across species and in other family C GPCRs, including mGluRs and *γ*-aminobutyric acid type B receptors.^26^, ^27^, ^28^

Previously, Bai et al^10^ reported a similar compensatory phenomenon for the human CaSR by comparing Ca^2+^_o_-induced Ca^2+^_i_ mobilization in HEK-293 cells that were transfected with either G143E (ECD) or A877X (proximal ICD), either alone or together. Although cells transfected with CaSR^G143E^ or CaSR^A877X^ alone were nonfunctional, cells cotransfected with these mutant subunits exhibited partially restored function. Our findings suggest that the same phenomenon can be observed, at least for G_q/11_-dependent Ca^2+^ mobilization, regardless of whether the intracellular mutation is located in ICL3 or the proximal C terminus. Recent findings from cryo-EM structural analysis of G_q_ protein–bound CaSRs provide an explanation in which ICL3 (residues 794–803) and proximal C-terminal segment helix-8 (residues I869 to F881) are both required for G_q_ binding.^15^^,^^16^

Having confirmed that CaSR_1_^S170A^:CaSR_2_^F801A^ mutant heterodimers exhibit partially restored function, we considered whether these responses might be enhanced by disabling ICD residue T888, the receptor’s primary PKC negative regulatory site.^7^^,^^8^ Therefore, we investigated the effect of introducing T888A to disable the inhibitory PKC site into either CaSR_1_^S170A^ or CaSR_2_^F801A^ mutant subunits and then coexpressed the subunits as appropriate to produce CaSR_1_^S170A/T888A^:CaSR_2_^F801A^, CaSR_1_^S170A^:CaSR_2_^F801A/T888A^, or CaSR_1_^S170A/T888A^:CaSR_2_^F801A/T888A^. Interestingly, we observed enhanced signaling from mutant heterodimers in which T888A was introduced into either CaSR_1_^S170A^ or CaSR_2_^F801A^, and the cells were transfected to generate CaSR_1_^S170A/T888A^:CaSR_2_^F801A^ and CaSR_1_^S170A^:CaSR_2_^F801A/T888A^. Even more interestingly, we observed substantial further enhancement of the signaling responses in mutant heterodimers in which T888A was introduced into the intracellular C termini of both mutant subunits (ie, to generate CaSR_1_^S170A/T888A^:CaSR_2_^F801A/T888A^). The changes in CaSR signaling could not be attributed to differences in total or surface expression of the various CaSR proteins (Fig. 1). Therefore, the data support the conclusion that the dimeric ICDs work independently to support Ca^2+^_o_-stimulated Ca^2+^_i_ mobilization and raise the possibility that under normal physiological conditions, the CaSR cycles between 2 primary activation states in which first one subunit and then the other binds to a single heterotrimeric G protein. If this concept is correct, phosphorylation of T888 on the G_bound_ subunit and dephosphorylation of T888 on the G_free_ subunit may be necessary requirements for the release of the G protein from subunit-1, adoption of the neutral symmetric dimer conformation, and transition to a new asymmetric conformation in which subunit-2 is configured for G protein binding (Supplemental Fig. 1). The inherent asymmetry of active G protein–bound dimeric CaSR structures, in which only one G protein molecule is bound at a time does not support a symmetric model of the MWC type but would be potentially closer to a sequential (KNF) model of receptor activation. Under circumstances in which neither subunit is available for phosphorylation, as in the case of CaSR_1_^S170A/T888A^:CaSR_2_^F801A/T888A^, it would seem plausible that the receptor would switch repeatedly between the active state of subunit-1 and the neutral state, but not the active state of subunit-2, which is unable to support G protein binding in the presence of F801A.

Previously, the CaSR intracellular C-terminal domain was shown to be required for G_q/11_-mediated activation of phosphoinositide phospholipase C, Ca^2+^_i_ mobilization, and extracellular signal-regulated kinase 1/2 (ERK_1/2_) phosphorylation but not G_i/o_-dependent inhibition of adenylyl cyclase.^11^^,^^26^^,^^29^^,^^30^ Upon activation, the CaSR’s VFT dimers appear to preferentially adopt a closed-closed configuration, ie, to operate symmetrically.^3^^,^^14^ However, CaSR homodimers couple to only one heterotrimeric G protein molecule at a time,^15^^,^^16^ consistent with reports for other class C GPCRs.^31^^,^^32^

Thus, in the process of activation, resting receptor symmetry is broken. In the subunit that binds the G protein, there is selective deformation of TM-6 followed by a closer apposition and reorientation of the 7-TMD dimer interface. At the same time, a shallow binding pocket for the C terminus of G-*α* forms dependent on ICL3 and the intracellular surface of TM3, with contributions from an extended loop from ICL2, which varies according to the species of G protein (eg, G_q/11_, G_i/0_, or G_s_), and residue K644 in ICL1. In the case of G_q/11_ but not G_i/o_, helix-8 of the ICD proximal C terminus also contributes.

Consistent with these observations, we found that the introduction of one T888A mutation, regardless of the subunit that received it, was sufficient to promote partially restored Ca^2+^_i_ mobilization from heterodimers formed from CaSR subunits containing either: (1) the mutation S170A, which disables VFT closure in homodimers; and (2) the mutation F801A, which disables G protein activation in homodimers (Fig. 3). Not immediately consistent with the results of the recent structural studies referred to above, however, we observed that the introduction of T888A mutations into both CaSR subunits substantially enhanced Ca^2+^_o_-induced Ca^2+^_i_ mobilization. Because the introduction of T888A did not restore Ca^2+^_o_-stimulated Ca^2+^_i_ mobilization in cells transfected singly with either CaSR_1_^S170A/T888A^, when compared with CaSR_1_^S170A^, or CaSR_2_^F801A/T888A^, when compared with CaSR_2_^F801A^, we conclude that in heterodimers formed from these subunits, T888 phosphorylation applies a critical brake on G protein coupling, and that elimination of T888 phosphorylation on both subunits maximizes available G protein docking time. Indeed, it leads us to speculate that maximal signaling output in WT homodimers requires a reaction mechanism in which first one and then the other subunit supports G protein binding, dependent upon a cycle of C-terminal phosphorylation between CaSR_1_ and CaSR_2_ subunits (Supplemental Fig. 1).

It is important to note that the experimental model did not allow us to investigate the CaSR’s requirements for 1 versus 2 sets of intracellular loops. In the event that maximal receptor-mediated signaling requires a phosphorylation-dependent cycle, in which one, and then the other, subunit engages in G protein binding and activation, it seems feasible that both sets of intracellular loops are normally required for maximal CaSR signaling.

In the present study, we also observed that the introduction of 1 or 2 intracellular C-terminal T888A gain-of-function mutations partially rescued Ca^2+^_i_ mobilization from the inactive CaSR_1_^S170A^ ECD mutant. S170A is nonfunctional up to high Ca^2+^_o_ concentrations (40 mM calcium), consistent with previous studies where no significant response was identified upon exposure of CaSR_1_^S170A^ to 50 mM calcium.^33^ S170A also exhibits reduced responsiveness to the L-amino acid activator L-Phe.^34^ Further, crystal structure analysis of the CaSR ECD demonstrates that Ser-170 contributes to L-Trp binding and adoption of the active closed state.^3^ The homologous residue in mGluR1, Thr-188, supports both glutamate-dependent and glutamate-independent VFT domain closure.^25^ Interestingly, loss of inhibitory phosphorylation in the intracellular C-terminal domain upon T888A mutation rescued the loss-of-function in CaSR_1_^S170A^. Indeed, Ca^2+^_o_-induced Ca^2+^_i_ responses in HEK-293 cells transfected to generate homodimers of the double mutant CaSR_1_^S170A/T888A^, closely resembled control Ca^2+^_o_-induced Ca^2+^_i_ responses in HEK-293 cells transfected with CaSR^WT^, provided that cells expressing mutant homodimers were also incubated in the presence of the PAM NPS R-568 (Fig. 6). Thus, R-568 can compensate for the impairment of VFT domain closure (S170A), but only if the inhibitory effect of Thr-888 is absent (as seen when also expressing T888A), whereas it cannot compensate for the loss of G protein coupling (F801A) regardless of Thr-888 removal.

Therefore, the ability of the CaSR to activate Ca^2+^_i_ mobilization is not solely dependent on an intact ECD because a disinhibited intracellular C-terminal domain can rescue CaSR function. In agreement with this result, previous studies have shown that CaSR mutants which lack the entire ECD retain Ca^2+^_o_ sensitivity in the presence of PAMs^35^^,^^36^ and that removal of the inhibitory phosphorylation sites in the so-called “headless” CaSR can substantially increase its ability to mobilize Ca^2+^_i_.^19^ Thus, novel treatments for inactivating mutations of the CaSR in humans might target inhibition of CaSR phosphorylation or activation of CaSR dephosphorylation.

In conclusion, the present study provides new insights into the role of the ICDs in homodimeric CaSR signaling via the Ca^2+^_i_ mobilization pathway. In particular, using a *trans*-activation model, we have found that one intracellular C-terminal domain is sufficient for receptor function but that both intracellular C-terminal domains are necessary for maximal Ca^2+^_o_-induced Ca^2+^_i_ mobilization.

## Conflict of interest

The authors declare no conflicts of interest.
