# Supplementary material for: Both calcium-sensing receptor intracellular C-terminal domains support homodimer signaling
Source: Mol Pharmacol. 2025 Sep 23;107(11):100077. doi: 10.1016/j.molpha.2025.100077 (PMC12799464; doi:10.1016/j.molpha.2025.100077)
Supplement: Supplementary Figure 1 [file mmc1.pdf]

Both Calcium-Sensing Receptor Intracellular C-terminal Domains support Homodimer Signalling

Lenah S Binmahfouz, Mahvash A Goolam, Eleanor Barker, Arthur D Conigrave and Donald T Ward

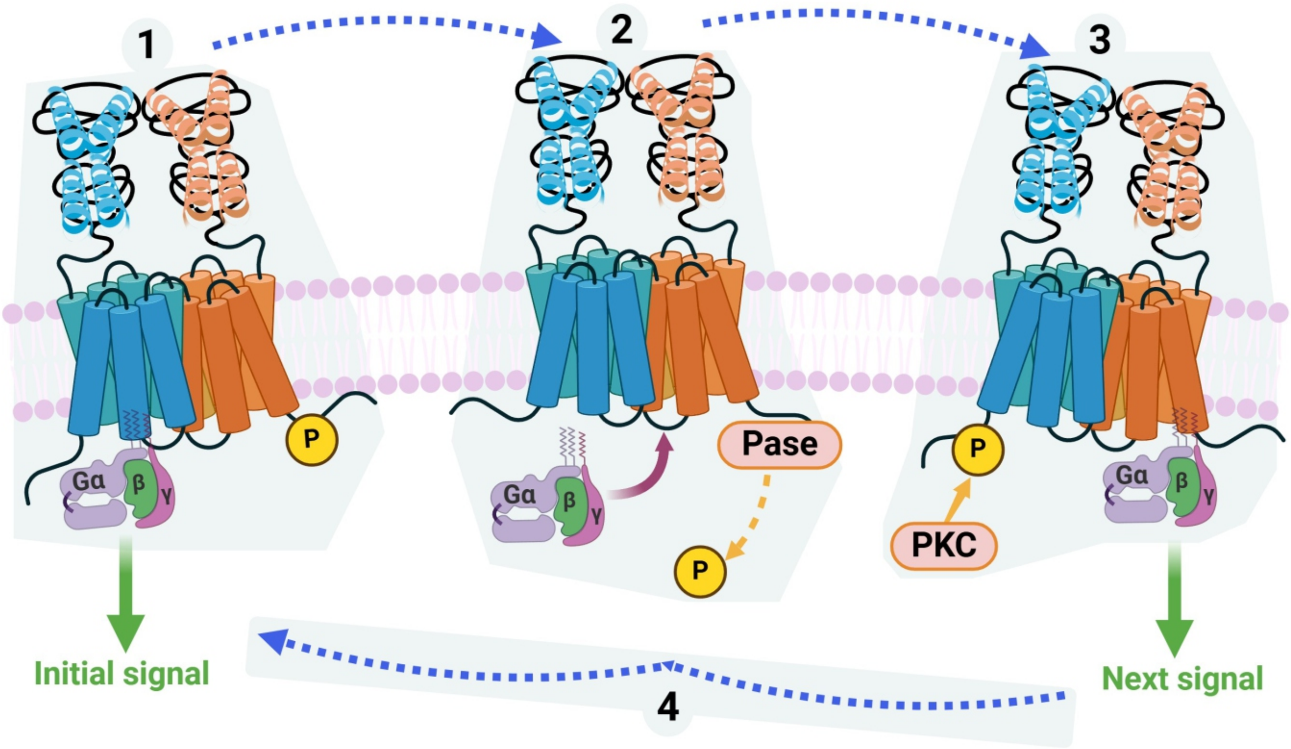

Supplemental Figure 1. Schematic representation of a proposed model for how CaSR phosphorylation might modulate G protein switching between CaSR monomers. Initially (Step 1), one CaSR monomer (blue) is coupled to the G protein while the other monomer (orange) is phosphorylated (most likely on T888) thus preventing switching, during which the coupled CaSR elicits Ca^{2+}_i mobilisation i.e. the upstroke of a single Ca^{2+}_i oscillation. Dephosphorylation of the site by a phosphatase (Pase) allows the G protein to switch to the other monomer (Step 2) during which there is no Ca^{2+}_i mobilisation i.e. the downstroke of the oscillation. Next, the first monomer becomes phosphorylated preventing the G protein from switching back (Step 3) permitting the next Ca^{2+}_i oscillation, until the next phase of dephosphorylation permits G protein switching back to the original state (Step 4).
